# Rapid oral transmucosal delivery of zaleplon–lavender oil utilizing self-nanoemulsifying lyophilized tablets technology: development, optimization and pharmacokinetic evaluation

**DOI:** 10.1080/10717544.2022.2115165

**Published:** 2022-08-28

**Authors:** Sarah A. Ali, Nabil A. Alhakamy, Khaled M. Hosny, Eman Alfayez, Deena M. Bukhary, Awaji Y. Safhi, Moutaz Y. Badr, Rayan Y. Mushtaq, Majed Alharbi, Bader Huwaimel, Mohammed Alissa, Sameer Alshehri, Ali H. Alamri, Taha Alqahtani

**Affiliations:** a Department of Oral Diagnostic Sciences, Faculty of Dentistry, King Abdulaziz University, Jeddah, Saudi Arabia; b Department of Pharmaceutics, Faculty of Pharmacy, King Abdulaziz University, Jeddah, Saudi Arabia; c Center of Excellence for Drug Research and Pharmaceutical Industries, King Abdulaziz University, Jeddah, Saudi Arabia; d Department of Oral Biology, Faculty of Dentistry, King Abdulaziz University, Jeddah, Saudi Arabia; e Department of Pharmaceutics, College of Pharmacy, Umm Al-Qura University, Makkah, Saudi Arabia; f Department of Pharmaceutics, College of Pharmacy, Jazan University, Jazan, Saudi Arabia; g Department of Pharmaceutics, College of Clinical Pharmacy, Imam Abdulrahman Bin Faisal University, Dammam, Saudi Arabia; h Department of Pharmaceutical Chemistry, Faculty of Pharmacy, King Abdulaziz University, Jeddah, Saudi Arabia; i Department of Pharmaceutical Chemistry, College of Pharmacy, University of Ha’il, Ha’il, Saudi Arabia; j Department of Medical Laboratory Sciences, College of Applied Medical Sciences, Prince Sattam bin Abdulaziz University, Al-Kharj, Saudi Arabia; k Department of Pharmaceutics and Industrial Pharmacy, College of Pharmacy, Taif University, Taif, Saudi Arabia; l Department of Pharmaceutics, College of Pharmacy, King Khalid University, Abha, Saudi Arabia; m Department of Pharmacology, College of Pharmacy, King Khalid University, Abha, Saudi Arabia

**Keywords:** Zaleplon, fast-disintegrating tablet, lavender oil, pharmacokinetics, mixture design

## Abstract

Based on the administration convenience, transmucosal buccal drug delivery allows special strength points over peroral routes for systemic delivery. It could achieve local or systemic effect and boost drugs’ bioavailability for agents with first pass metabolism. The current study aimed to manufacture and optimize a lavender oil–based nanoemulsion loaded with zaleplon and incorporate it into fast-disintegrating tablets to promote its dissolution and oral bioavailability via oral mucosa. Zaleplon-loaded nanoemulsions were devised with various levels of lavender oil (10% to 25%), the surfactant Sorbeth-20 (35% to 65%), and the co-surfactant HCO-60 (20% to 40%); the extreme vertices mixture statistical design was adopted. The droplet size and drug-loading efficiency were the evaluated. The optimal formulation was transformed into self-nanoemulsified lyophilized tablets (ZP-LV-SNELTs), which were tested for their uniformity of content, friability, and disintegration time with in-vitro release. Finally, the pharmacokinetic parameters of the ZP-LV-SNELTs were determined and compared with those of marketed formulations. The optimal nanoemulsion had a droplet size of 87 nm and drug-loading capacity of 185 mg/mL. ZP-LV-SNELTs exhibited acceptable friability and weight uniformity and a short disintegration time. The in-vitro release of ZP-LV-SNELTs was 17 times faster than that of the marketed tablet. Moreover, the optimal ZP-LV-SNELTs increased the bioavailability of zaleplon in rabbits by 1.6-fold compared with the commercial tablets. Hence, this investigation revealed that ZP-LV-SNELTs delivered zaleplon with enhanced solubility, a fast release, and boosted bioavailability thru oral mucosa which provided a favorable route for drug administration which is suggested to be clinically investigated in future studies

## 1. Introduction

Oral mucosa covering oral cavity is known to be formed of three distinctive layers, namely, the epithelium which lines the cavity, supporting basement membrane, and finally connective tissues (Chinna Reddy, 2011). The mechanism of drug absorption via buccal mucosa embraces two main pathways which are intercellular and intracellular routes. The drug transport potential of oral mucosa is affected by drug’s molecular weight, degree of lipophilicity and the pH of the surrounding medium (Nielsen & Rassing, 1999). Buccal route offers an attractive alternative administration pathway for sundry of drugs that cannot be efficiently delivered thru traditional oral route, due the pre-systemic clearance they may extensively encounter in liver (Shojaei et al., 2001). Transmucosal buccal drug delivery allows special strength points over peroral routes for systemic delivery. Such advantages encompass the excellent accessibility, quite large area of smooth muscles and comparatively stable and rapidly recovered mucosa, therefore convenient for applying controlled release formulations (Hua, 2019). Further, buccal route gains a great patient acceptability and compliance relative to other non-oral transmucosal routes. Additionally, this route offers a straightway delivery to blood by means of the internal jugular vein, thus eschews acid hydrolysis in the G.I.T and obviates drugs’ first pass metabolism in liver yielding considerable increase in bioavailability (Indiran Pather et al., 2008).

Zaleplon (ZP) is a fast-acting hypnotic drug of the pyrazolopyrimidine class with a mild adverse-effect profile. It is primarily recommended for the short-term management of insomnia (Ebbens & Verster, 2010; Manda et al., 2018). Insomnia may be described as frustration with sleep quality or quantity, trouble starting or maintaining sleep, and repeated nighttime awakenings. It affects 15% to 30% of the population at some point in their lives and is associated with depression, psychiatric illnesses, several other diseases and has significant socioeconomic consequences and is associated with a diminished quality of life (Buysse et al., 2008; Foda & Bakhaidar, 2010; Hosny & Banjar, 2013; Qaseem et al., 2016; Sakhare, 2017). Neither rebound insomnia nor dependence was observed on discontinuing zaleplon. However, sustained hypnotic efficacy was often achieved (Popescu, 2015; Dudhipala, 2016; Vermeeren et al., 2017).

The therapeutic efficiency of ZP as a benzodiazepine-like agent has been documented. This is due to ZP’s interaction with the receptor of gamma-aminobutyric acid type A (GABA_A_), preferentially at the α_1_β_2_γ_2_ subunit binding site, which is the benzodiazepine binding site in the central nervous system. Thus, it is also used as a strong anticonvulsant medication in pentylenetetrazole and electroshock-induced convulsions (Hosny et al., 2006; Abd-Elrasheed et al., 2018).

The oral ingestion of ZP has several drawbacks, such as the massive first-pass hepatic metabolism of the drug after gastrointestinal absorption, with a limited absolute bioavailability of 30%. This is due to its poor dissolution as a result of poor aqueous solubility and the resulting delays in its onset of action (De Jong & Borm, 2008; Naahidi, 2013).

Nanosized paradigms are emerging tools that are used to address issues of the delivery of poorly bioavailable drugs. Importantly, considerable research is now being devoted to the use of nanotechnology in targeting and improving the release of a variety of active therapeutic ingredients, providing enhanced absorption and bioavailability (Ochekpe et al., 2009; Park, 2013; Singh et al., 2017).

Nanoemulsions (NEs), also known as submicron emulsions, miniemulsions, and ultrafine emulsions, have become increasingly attractive options in terms of dosage form designs and pharmacotherapies. These nanoscale entities serve primarily as vehicles for the delivery of therapeutics, in particular, poorly soluble drugs that are prone to hydrolysis (De Jong & Borm, 2008). NEs are biodegradable, biocompatible, optically clear, and easy-to-produce emulsions (Shafiq-un-Nabi et al., 2007; Bhatt & Madhav, 2011). They have a nanoscale droplet size averaging between 20 and 200 nm. Besides having good solubilization, they improve gastrointestinal absorption and lower inter- and intra-subject variability for diverse bioactives and possess considerable thermodynamic stability. This is due to their small droplet size, which decreases their gravitational force and enhances their Brownian motion to affect gravity, therefore preventing creaming or sedimentation in suitable storage conditions. NEs are frequently made by combining two non-miscible fluids, such as to form a single phase that can then be stabilized applying emulsifying agents, such as a mixture of a surfactant and a co-surfactant which will reduce the tension between the two immiscible phases’ interfaces (Cui et al., 2009; Shah et al., 2010; Lovelyn & Attama, 2011; El-Say et al., 2017).

The use of NEs in the pharmaceutical industry is especially promising; a number of patents have been submitted for NE formulations, but many of these NEs have not been marketed yet (Tiwari et al., 2006). Cui et al., for example, created a unique self-microemulsifying drug delivery system that successfully increased curcumin solubility and oral absorption (Zülli et al., 2006). Similarly, previous studies have reported that the o/w NEs containing the hydrophobic anticancer drug paclitaxel overcame the drug’s low oral bioavailability. They used peanut oil as the internal oil phase, egg lecithin as the principal emulsifier, and water as the exterior phase (Zidan et al., 2015). Ubiquinone, also known as Coenzyme Q10 (CoQ10), is a naturally occurring substance in the body; it is utilized for the production of energy within cells and acts as an antioxidant agent. CoQ10 is also available as a dietary aid. In this form it may have the major drawback of low oral bioavailability as a result of its high lipophilicity. A recent study revealed the significant enhancement of the bioavailability of CoQ10 following its encapsulation in NEs. There was even more improvement with NEs that contained tocopherol and CoQ10 in separate nanodroplets (Chen et al., 2015).

Nowadays, oral drug delivery systems composed of drug-loaded nanosized carriers can be preferred route of drug administration. In particular, nanoentities in solid forms have been of interest (Pabari & Ramtoola, 2012). Fast-disintegrating tablets (FDTs), also known as orodispersible tablets (ODTs), are an alternative to traditional oral capsules and tablets (Chinwala, 2020). The Center for Drug Evaluation and Research of the United States Food and Drug Administration described the ODT as ‘a solid dosage form containing medicinal substances which disintegrates rapidly, usually within a matter of seconds, when placed upon the tongue’ in the Orange Book (Ghourichay et al., 2021). ODTs are ‘uncoated tablets intended to be placed in the mouth, where they disperse rapidly before being swallowed’, according to the European Pharmacopeia, and they should dissolve within 3 minutes (Sharma, 2013). FDTs contain superdisintegrants, which facilitate water uptake into tablets; they are a prerequisite for disintegration. Disintegration of FDTs occurs rapidly in a small volume of saliva; drinking water is not necessary for disintegration of these tablets (Rao et al., 2012; Nafady, 2014). FDTs have an improved dissolution rate. They also have a rapid onset of action because absorption occurs directly in the mouth, before gastric absorption; hence, the amount of the active agent exposed to first-pass hepatic metabolism is decreased as opposed to that of conventional dosage forms. Altogether, these features ultimately increase the bioavailability of drugs. This form of drug delivery is gaining wider popularity day by day due to its numerous advantages (Alhakamy & Hosny, 2019). The main goal of the current investigation was to develop and optimize lavender oil–based nanoemulsions loaded with zaleplon and incorporate the optimum formulation into fast-disintegrating tablets to boost the bioavailability and onset of action of the ZP and administering the developed optimal paradigms via the advantageous transmucosal buccal route.

## 2. Materials and method

### 2.1. Materials

Zaleplon, gelatin, microcrystalline cellulose (Avicel PH-101), and silica fume (0.007 mm) were purchased from Sigma-Aldrich (St. Louis, MO, USA). Lavender oil (LV) was obtained from Acros Organics (Geel, Belgium). D-Mannitol was attained from Winlab, Ltd. (Leicestershire, UK). Propylene glycol, Cremophor EL, and sodium carboxymethyl were obtained from Spectrum Chemical Manufacturing Corporation (Gardena, CA, USA). Polacrillin potassium (KYRON T-314) was obtained as a gift from Glenmark Generics, Ltd. (Mumbai, India). Polyethylene glycol-40, −50, and −60 hydrogenated castor oil (HCO-40, HCO-50, HCO-60), polyethylene glycol-25-stearate (MYS-25V), polyoxyethylene lanolin (Sorbeth-20), polyoxyethylene-C21-ethers (Laureth-21), and sefsol were purchased from Nikko Chemicals Co., Ltd. (Tokyo, Japan). A Marketed zaleplon tablet (Sonata) was acquired from Pfizer Ltd. (UK). All other chemicals and reagents used were of analytical grade.

### 2.2. Method

#### 2.2.1. Solubility studies

The solubility of ZP in various co-surfactants and surfactants was determined in hopes of finding a variety of self-emulsifying regions that could aid in the development of formulations containing ZP and LV. By dispersing an additional quantity of ZP in 5 mL of each solution individually, the solubility of ZP in several surfactants (Cremophor EL, Laureth-21, MYS-25V, and Sorbeth-20) and co-surfactants (propylene glycol, HCO-60, HCO-40, and HCO-50) was determined. Then, the formulations were placed for 72 h in a water bath at 25 ± 2 °C (Model 1031; Gujarat Fluorochemicals GmbH, Hamburg, Germany). The mixtures were centrifuged (Sigma 3k30, Osterode am Harz, Germany) at 4500 rpm for 15 min after equilibrium of the mixtures was obtained. The supernatants were diluted with methanol. The drug concentration was 240 nm λmax utilizing a previously reported high-performance liquid chromatography (HPLC) method (Hosny & Rizg, 2018). The column used was the Symmetry C8, 5.0 µm column. The mobile phase contained acetonitrile (v/v), 0.02 M potassium dihydrogen phosphate (w/v), and methanol (v/v) in a ratio of 25:60:15. The flow rate was 1.0 mL/min.

#### 2.2.2. Strength of ZP-LV emulsifying regions in selected surfactants and co-surfactants

The existence of a self-emulsifying region was determined utilizing a pseudoternary phase diagram, which predicted the higher and lower amounts of each component of the self-nanoemulsified drug delivery systems (SNEDDS), which had been chosen based on solubility. When the mixture of the ZP, LV, co-surfactant, and surfactant was stirred gently, a transparent nanosized globule emulsion was produced. Therefore, the diagram was thought to be valuable for determining the various amounts of each component of the SNEDDS.

The produced mixtures were evaluated for droplet size uniformity and distribution using the Zetatrac instrument (Microtrac MRB, Montgomeryville, PA, USA). The NE regions could be recognized in the phase diagram when the droplet size was smaller than 1 μm. The NE formulations to be loaded with ZP were then chosen.

#### 2.2.3. Statistical design for preparation of ZP-LV-SNEDDS

A mixture statistical design was proposed for the production and optimization of the NE formulations due to its accuracy and effectiveness (Hosny et al., 2016; El-Say et al., 2017; Bakhaidar et al., 2022). The excessive vertices mixed design was chosen for the development of the suggested 18 NE formulations to assess and optimize the influence of the independent variables, which were the percentages of LV (A), surfactant Sorbeth-20 (B), and co-surfactant HCO-60 (C); the percentages were 10% to 25%, 35% to 65%, and 20% to 40%, respectively. To synthesize the ZP-LV-SNEDDS with the smallest globule size (Y_1_) and highest drug-loading capacity (Y_2_), combinations with various percentages of the three independent variables were utilized. The responses were allocated, and the formulations were optimized using the design of experiment. Table 1 shows the various elements and their fractions that were chosen for NE optimization.

**Table 1.** Selected ratio of variables for the mixture design.

Component	Ratio	
	Low	High
A: Lavender oil %	10.0	25.0
B: Sorbeth-20%	35.0	65.0
C: HCO-60%	20.0	40.0

#### 2.2.4. Visual prediction of self-emulsification

The efficiency of the ZP-LV-SNEDDS formulations was determined via visual observation of their degree of clarity and spontaneous emulsification, along with any sign of instability, such as coagulation or cracking (17).

#### 2.2.5. Determination of globule size (Y_1_)

Under continuous stirring, NE samples (0.1 mL) were diluted using double-distilled deionized water (10 mL). The dynamic light-scattering approach was utilized to assess the droplet size and polydispersity index (PDI) of the developed formulations (Zetatrac instrument, Microtrac MRB, Montgomeryville, PA, USA) (Hosny et al., 2006).

#### 2.2.6. Determination of drug-loading capacity (Y_2_)

The ZP-loading capacity of the NE formulations was assessed by placing certain known quantities of ZP in vials containing 1 mL of each plain NE formulation. The vials were kept for 24 h in a water bath at a temperature of 25 ± 2 °C until equilibrium was reached. After reaching equilibrium, the mixtures were centrifuged at 4500 rpm for approximately 15 min. To evaluate the loading capacity for each SNEDDS combination, the supernatants were collected and diluted with methanol, and the ZP concentration was measured at a λ_max_ of 240 nm using the HPLC technique described in Section 2.2.1. The drug-loading capacity (Y_2_) was determined using the following equation:

(1) Drug−loading capacity = (1–Drug content in the product obtained (mg)/Total product weight (mg)) × 100

Components and the observed mean globule size (Y_1_) and drug-loading capacity (Y_2_) of various ZP-LV-SNEDDS by mixture design are depicted in Table 2.

**Table 2.** Components and observed mean globule size (Y_1_) and drug-loading capacity (Y_2_) of various ZP-LV-SNEDDS by mixture design.

Run	A:Lavender oil	B:Sorbeth-20	C:HCO-60	Y_1_: Globule size (nm)	Y_2_: Loading capacity (mg/mL)	PDI
1	0.100	0.500	0.400	73 ± 2.1	120 ± 2.1	0.24
2	0.100	0.639	0.260	65 ± 0.9	105 ± 0.8	0.10
3	0.170	0.428	0.400	99 ± 3.4	181 ± 3.9	0.26
4	0.150	0.650	0.200	80 ± 2.5	51 ± 1.0	0.31
5	0.100	0.564	0.335	71 ± 3.0	135 ± 1.5	0.19
6	0.240	0.405	0.350	127 ± 4.8	182 ± 3.6	0.27
7	0.170	0.540	0.290	98 ± 3.9	130 ± 4.2	0.22
8	0.180	0.512	0.306	101 ± 2.9	145 ± 5.1	0.30
9	0.150	0.594	0.251	85 ± 0.9	94 ± 2.1	0.33
10	0.230	0.516	0.255	113 ± 5.1	96 ± 1.9	0.18
11	0.100	0.639	0.260	66 ± 3.3	104 ± 1.7	0.13
12	0.250	0.350	0.400	143 ± 2.5	198 ± 2.6	0.32
13	0.180	0.512	0.306	102 ± 1.9	152 ± 3.3	0.17
14	0.170	0.428	0.400	100 ± 0.8	186 ± 4.5	0.29
15	0.250	0.550	0.200	120 ± 4.5	130 ± 4.0	0.11
16	0.250	0.454	0.295	127 ± 3.8	137 ± 3.0	0.20
17	0.180	0.512	0.306	100 ± 4.1	153 ± 2.7	0.33
18	0.200	0.599	0.200	103 ± 1.3	70 ± 1.0	0.26

#### 2.2.7. ZP-LV-SNEDDS optimization

The statistical program StatGraphics Centurion XV software, version 13.2.05, and a regression equation were utilized to link the variables with the measured responses (StatPoint Medical, Baltimore, MD, USA). The degrees of freedom F-ratio and *p*-value of all the variables and their interactions were stratified for an analysis of variance (ANOVA) of the determined responses’ models and for choosing the model that best fit the gathered data. A *p*-value of less than .05 was considered significant for the models. The components of the optimized ZP-LV-SNEDDS formulation were determined. Following optimization, the formulation (50 mL) was produced and assessed for droplet size and drug loading and then formulated into a lyophilized tablet.

#### 2.2.8. Preparation of ZP-LV-SNELTs

The optimized ZP-LV-SNEDDS was formulated into lyophilized tablets using several additives according to a previously published method (43-45). The pharmaceutical excipients utilized in the zaleplon–lavender oil–self-emulsifying lyophilized tablets (ZP-LV-SNELTs) were 2% porous fumed silica and 2% microcrystalline cellulose (Avicel) as adsorbents, 1.5% KYRON T-314 as a superdisintegrant, 0.5% sodium carboxymethyl cellulose, and 2% gelatin, and 1% mannitol was used as a structure-forming excipient. Briefly, a specified amount of the optimized ZP-LV-SNEDDS containing an amount of ZP sufficient for a 50 tablet was mixed with 50 mL of a gelatin solution (2% w/v) with a magnetic stirrer. The remaining substances were added with continous homogenization at 300 rpm until a homogenous dispersion was formed. The formed dispersions were emptied into a pocket blister pack, which was subjected to a lyophilization process to produce ZP-LV-SNELTs containing 10 mg of ZP in each individual tablet. The lyophilized tablets were kept at room temperature in a desiccator for further evaluation.

#### 2.2.9. In-vitro characterization of ZP-LV-SNELTs

The ZP-LV-SNELTs were evaluated according to the method described in USP 28/NF23 with regard to different factors, including weight uniformity, thickness, visual appearance, and drug content, in order to confirm the uniformity of the manufactured tablets (Alhakamy et al., 2022). Further, the in-vitro disintegration time and in-vitro dissolution tests were performed in order to confirm how quickly the tablets disintegrated and dissolved in conditions similar to those of the oral cavity. The dissolution test was performed for the ZP-LV-SNELTs in comparison with a commercially available ZP tablet, Sonata, using USP dissolution test apparatus II in 250 mL of simulated saliva fluid at pH 6.8.

#### 2.2.10. In-vivo bioavailability and pharmacokinetic study

##### 2.2.10.1. Selection of animals

This study was performed according to the institutional guidelines of the Animal Ethics Committee of Cairo Agriculture for Experimental Animals, Cairo, Egypt, Approval No. (111-03-22). In the in-vivo investigation, 12 albino male rabbits weighing between 2 and 2.5 kg were employed. The animals were fasted for 12 hours before the start of the experiment, and they were split into two groups of six rabbits each. The commercially available ZP tablet (reference standard) was given to group 1 at a dose of 1 mg/kg, and group 2 received the optimized ZP-LV-SNELTs at the same dose. Blood samples (1 mL) were withdrawn from the rabbits’ ear veins before drug administration and then again after 5, 15, 30, 45, and 60 min, as well as after 1.5, 2, 3, 4.5, 6, 9, 12, and 24 h. The samples were kept at 20 °C for further analysis.

##### 2.2.10.2. Pharmacokinetic analysis

A plasma concentration-time curve was constructed, and the data were fed into the WinNonlin Nonlinear Estimation Program to determine the maximum serum concentration (C_max_), time to peak drug concentration (T_max_)_,_ half-life (t_1/2_), and mean residence time (MRT). The linear trapezoidal rule was used to determine the area under the curve (AUC_0–24_). The relative bioavailability and the AUC_0–∞_ of the optimized ZP-LV-SNELTs were also calculated. Pharmacokinetic parameters of the optimized preparation and the reference tablet were statistically compared using the one-way ANOVA at a significance level of *p*-value less than 0.05 using an SPSS statistical program.

## 3. Results and discussion

### 3.1. Solubility studies

**Figure 1.**
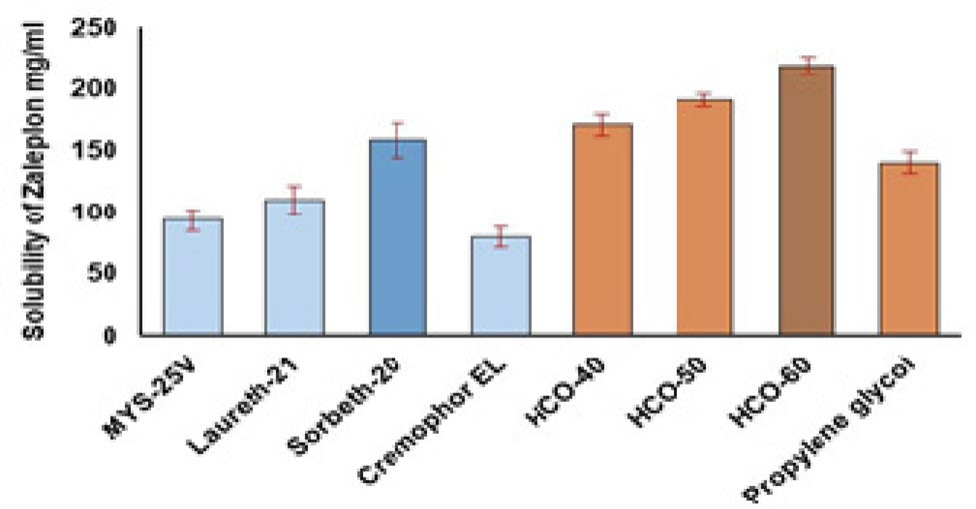
Solubility of ZP in various surfactants and co-surfactants.

The findings of ZP solubility studies on different surfactants and co-surfactants showed that the tested therapeutic agent had the highest solubility in the surfactant Sorbeth-20 and co-surfactant HCO-60. Figure 1 shows the associations between the percentages of solubilized ZP and the different surfactants and co-surfactants. Sorbeth-20 and HCO-60 solubilized the highest amount of ZP and were accordingly chosen for the construction of the pseudoternary phase diagram to assess the lower and upper limits of each of the NE components.

### 3.2. Strength of ZP-LV emulsifying regions with selected surfactants and co-surfactants

The emulsification procedure included mixing the aqueous and oily phases and then stabilizing the formed droplets using a mixture of surfactants and co-surfactants (Al-Amodi et al., 2020). Nonpolar hydrophobic compounds containing hydrocarbons and natural triglycerides are the most frequently used oil phase components; the aqueous phase is frequently made of solutes and electrolytes solubilized in water (Chen et al., 2015). In the present study, a pseudoternary phase diagram was developed to obtain the most precise concentration ranges of oil, surfactant, and co-surfactant that could produce the most stable NE regions.

**Figure 2.**
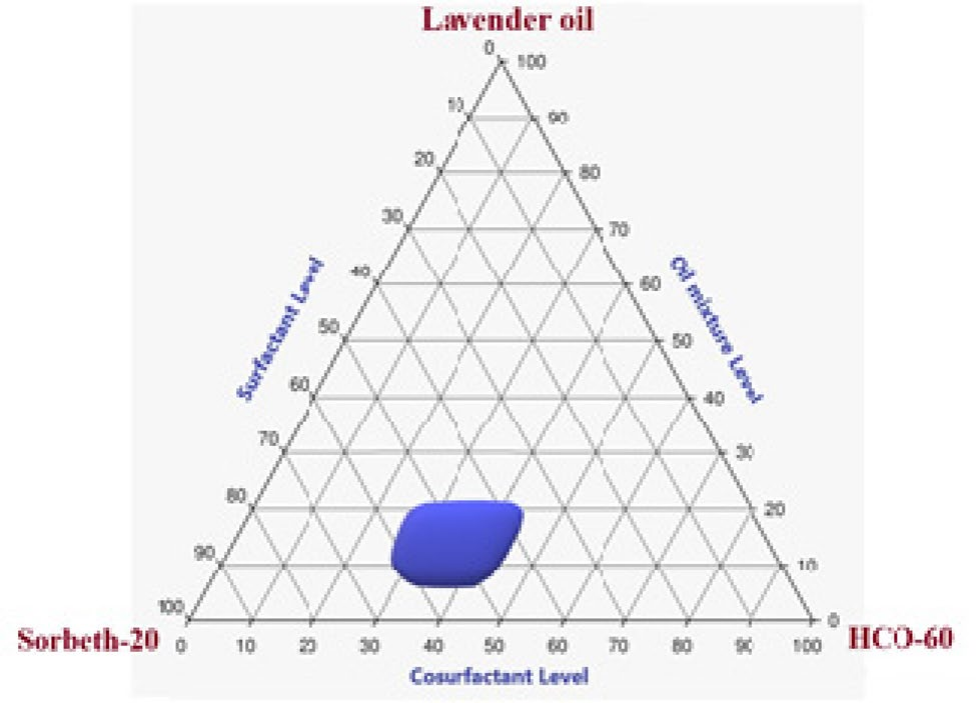
Pseudoternary phase diagram of LV, Sorbeth-20 surfactant, and HCO-60 co-surfactant.

As can be seen in Figure 2, the precise concentration ranges that could be used in the NE of LV, Sorbeth-20, and HCO-60 were 10% to 25%, 35% to 65%, and 20% to 40%, respectively. Consequently, these concentration ranges were adopted in the building of the statistical design used for the development and optimization of various ZP-LV-SNEDDS.

### 3.3. Visual appearance of ZP-LV-SNEDDS

The visual inspection of the ZP-LV-SNEDDS formulations revealed clear transparent dispersions with no turbidity or cracking. This showed the accuracy of the employed concentration ranges of the examined components and the formation of stable NEs.

### 3.4. Measurements of droplet size (Y_1_)

The droplet size of the NEs fluctuated between 65 ± 0.9 and 143 ± 2.5 nm, as shown in Table 2, with PDIs from 0.1 to 0.33 revealing the favorable stability, accepted homogeneity, and reasonable size distribution of the manufactured formulations.

A special quadratic model of polynomial analysis was selected by the experimental design to assess the significance of the percentages of LV (A), Sorbeth-20 (B), and HCO-60 (C) and their effects on the droplet size of the ZP-LV-SNEDDS. The chosen model had an adjusted R^2^ value of 0.9939; this was in line with the expected R^2^ value of 0.9452, as illustrated in Table 3. Data analysis by ANOVA yielded the following equation:

(2) $$\mathrm{Droplet}\;\mathrm{size}=+170A+55.61B+55.62C+63.47\text{AB}+151.53\text{AC}+66.37\text{BC}–415.53A^{2}BC–94.56AB^{2}C–421.05ABC^{2}$$

**Table 3.** Regression analysis results for Y_1_ and Y_2_ responses.

Dependent variables	R^2^	Adjusted R^2^	Predicted R^2^	p-Value	F-value	Adequate precision
Y_1_	0.9968	0.9939	0.9452	0.0001	348.38	62.9238
Y_2_	0.9947	0.9899	0.9311	0.0001	209.20	52.2940

It was observed that all of the investigated variables had a significant effect on the droplet size of the NEs at a *p*-value of less than .0001. Interestingly, parameter A (i.e., percentage of LV) had the greatest effect on the droplet size because it gained the highest coefficient (170) when compared with Sorbeth-20 (55.61) and HCO-60 (55.62). Further, the interaction terms of parameter (A) attained higher coefficient values compared with the interaction terms that did not include factor (A).

**Figure 3.**
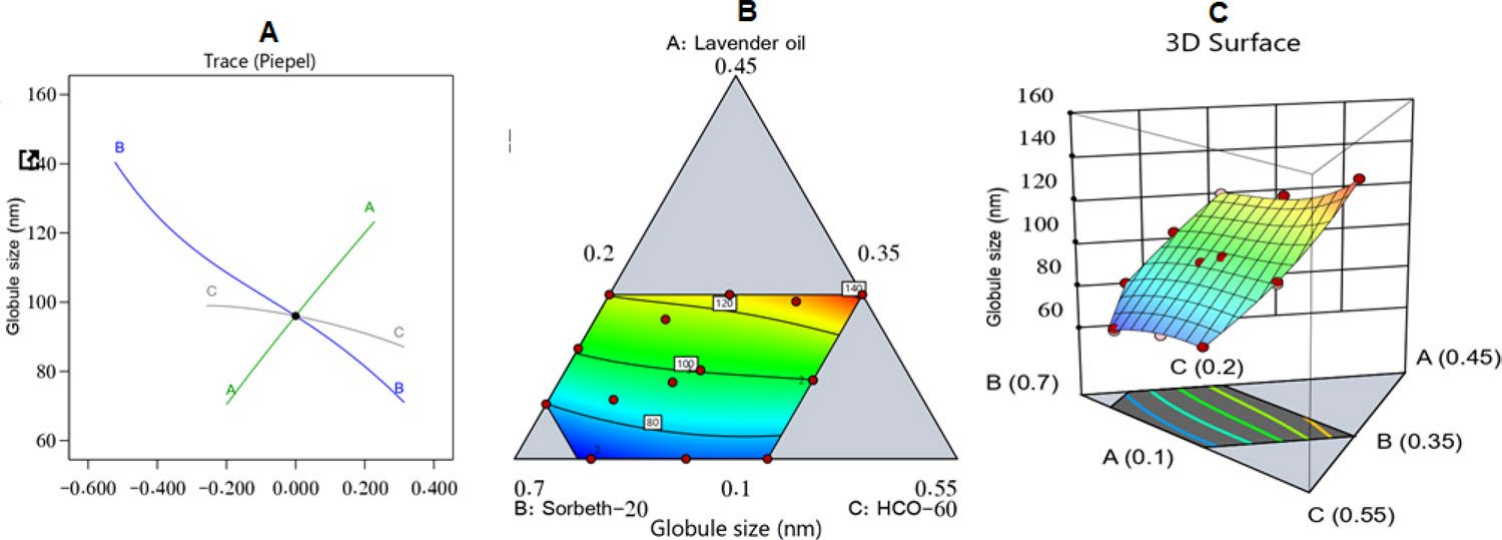
Main effect diagram (A), contour plot (B), and 3D surface plot (C) showing the effects of different independent variables on the droplet size of different ZP-LV-SNEDDS.

The increase in droplet size of the ZP-LV-SNEDDS with the increase in percentage of LV might be ascribed to the expected decrease in amounts of surfactant and co-surfactant used, which would decrease the ability of surfactant and co-surfactant to lower the droplet size and lead to the formation of larger droplets. Comparable outcomes were previously reported (Hosny et al., 2021; Md et al., 2021). Figure 3 shows the three-dimensional (3D) surface and contour plots in addition to the main effect diagram, which indicated the independent variables’ influence on the droplet size of the ZP-LV-SNEDDS. This clarified that the droplet size of the developed formulations was greatly affected by all of the studied factors.

Figure 3(A) shows that the measured droplet size was much decreased when the percentages of surfactant and co-surfactant were increased. This could be due to their ability to lower the interfacial tension between the aqueous and organic phases, thus developing droplets with smaller diameters (Rizg et al., 2022).

### 3.5. Evaluation of drug-loading capacity of ZP-LV-SNEDDS (Y_2_)

ZP loading in the NEs ranged between 51 ± 1.0 and 198 ± 2.6 mg/mL, as shown in Table 2.

A special quartic model of polynomial analysis was chosen to analyze the drug-loading data. The elected statistical design uncovered the efficacy of the model in evaluating the impact of the percentages of LV (A), Sorbeth-20 (B), and HCO-60 (C) on the ZP-LV-SNEDDS’ capacity to contain the drug. The model had an adjusted R^2^ value of 0.9899 and predicted R^2^ value of 0.9311; these values were in close accordance, as shown in Table 3. Data were analyzed using the one-way ANOVA and yielded the following equation:

(3) $$\mathrm{Drug}\;\mathrm{loading}\;\mathrm{capacity}\;=\;+\;660.20A+50.84B–39.02C–747.90\text{AB}–258.98\text{AC}+492.53\text{BC}–9428.64A^{2}BC+725.77AB^{2}C+7229.06ABC^{2}$$

As can be seen from the equation, all of the examined variables had a significant effect on the drug loading at a *p*-value of less than .0001.

However, the percentage of LV had the greatest effect on the drug loading; it acquired the highest coefficient (660.20) when compared with Sorbeth-20 (50.84) and HCO-60 (39.06). Additionally, the interaction terms containing factor (A) had higher coefficient values than interaction terms that did not contain factor (A).

**Figure 4.**
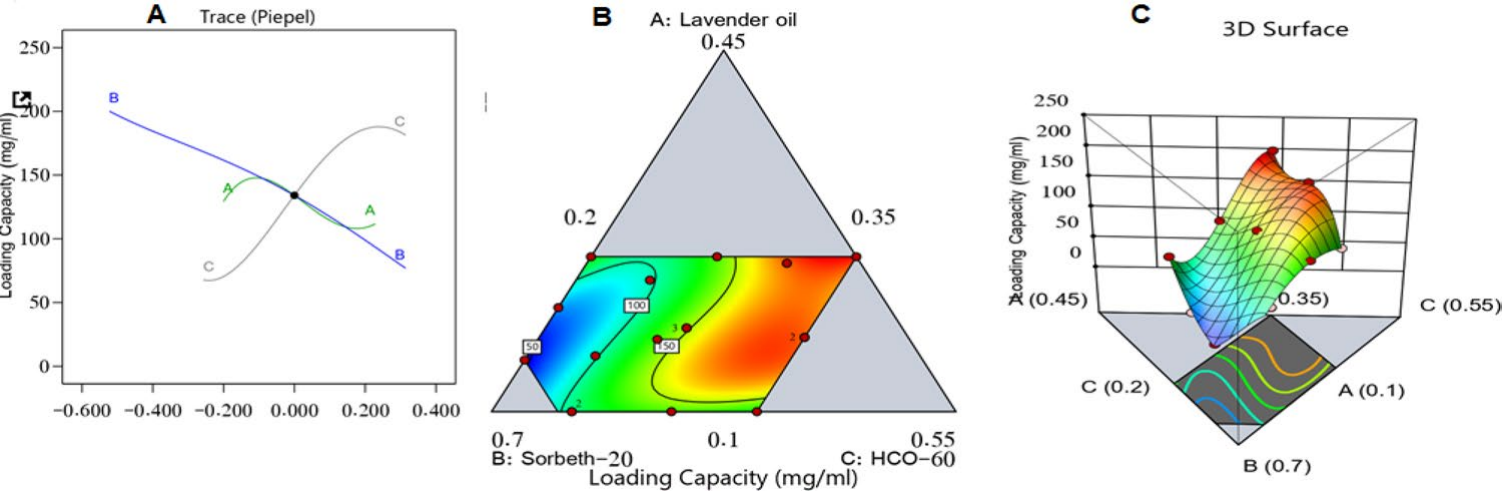
Main effect diagram (A), contour plot (B), and 3D surface plot (C) showing the effects of different independent variables on the drug-loading capacity of different ZP-LV-SNEDDS.

Figure 4 shows the influence of the independent variables on the ZP loading capacity in a main effect diagram and contour and 3D surface plots of the response.

The increase in drug-loading capacity due to the increase in percentage of LV could be due to the high affinity of the hydrophobic drug ZP for the organic phase; hence, the higher the percentage of oil, the more space available to accommodate the drug. It can also be seen from the figure that increasing the percentage of Sorbeth-20 decreased the drug-loading capacity of the formulations. This could be ascribed to the amphiphilic nature of factor B, which contributed to its ability to decrease the interfacial tension between the aqueous and organic phases, which might lead to the escape of the loaded drug into the surrounding aqueous phase similar reports were found in literature (Hosny et al., 2021; Md et al., 2021; Rizg et al., 2022).

### 3.6. Optimization of ZP-LV-SNEDDS

**Figure 5.**
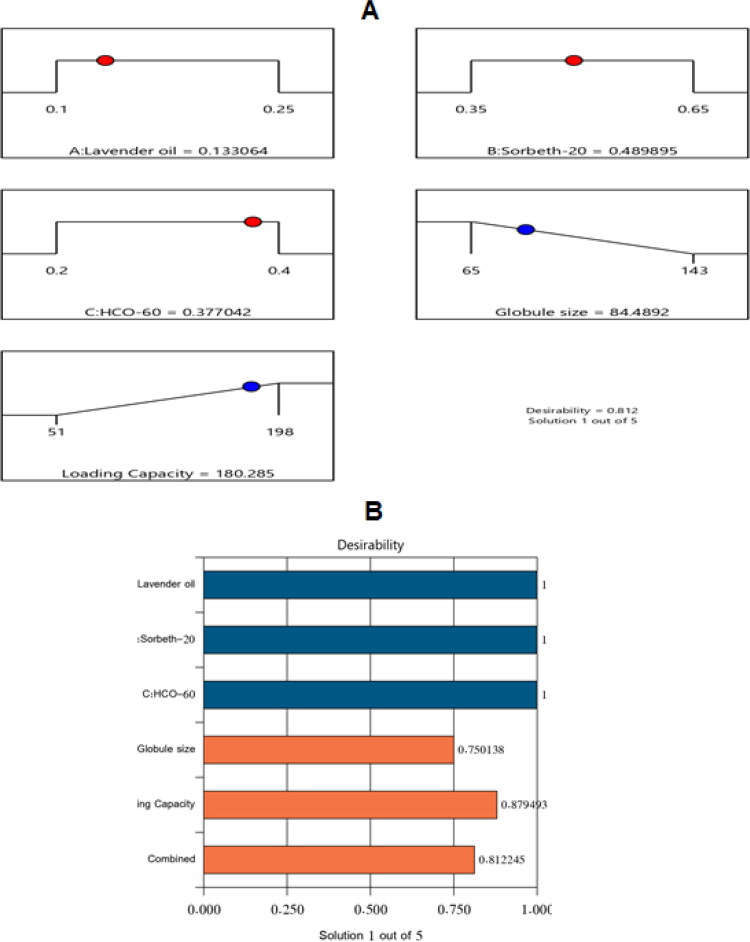
Bar chart and desirability ramp for optimization process. The desirability ramp illustrates the levels of factors and expected values for the dependent variables of the optimized ZP-LV-SNEDDS (A). The bar chart illustrates the values of desirability for the conjugated responses (B).

By examining the data, an optimal NE formulation was manufactured with the most suitable specifications. The utilized software offered diverse solutions using many combinations of amounts of the variables. The optimum formulation consisted of 13.3% LV, 49.0% Sorbet-20, and 37.7% HCO-60. The optimal formulation had a droplet size of 84.48 nm and a drug-loading capacity of 180.28 mg/mL, with a desirability of 0.812. Figure 5 shows a desirability ramp and bar chart with the factors’ levels and predicted values of the measured responses of the optimum formulation. Table 4 affirms that the experimental and predicted values of the optimal formulation’s parameters were closely related with no significant differences (*p* > .05), and this confirmed the precision and validity of the chosen models.

**Table 4.** Actual and experimental values of the optimized NE formulation.

Solution	LV oil %	Sorbeth-20%	HCO-60%	Droplet size (nm)	Drug loading (mg/mL)	Desirability
Predicated value	13.3	49.0	37.7	84.48	180.28	0.812
Experimental value	13.3	49.0	37.7	87	185	0.812

### 3.7. Check point analysis

**Figure 6.**
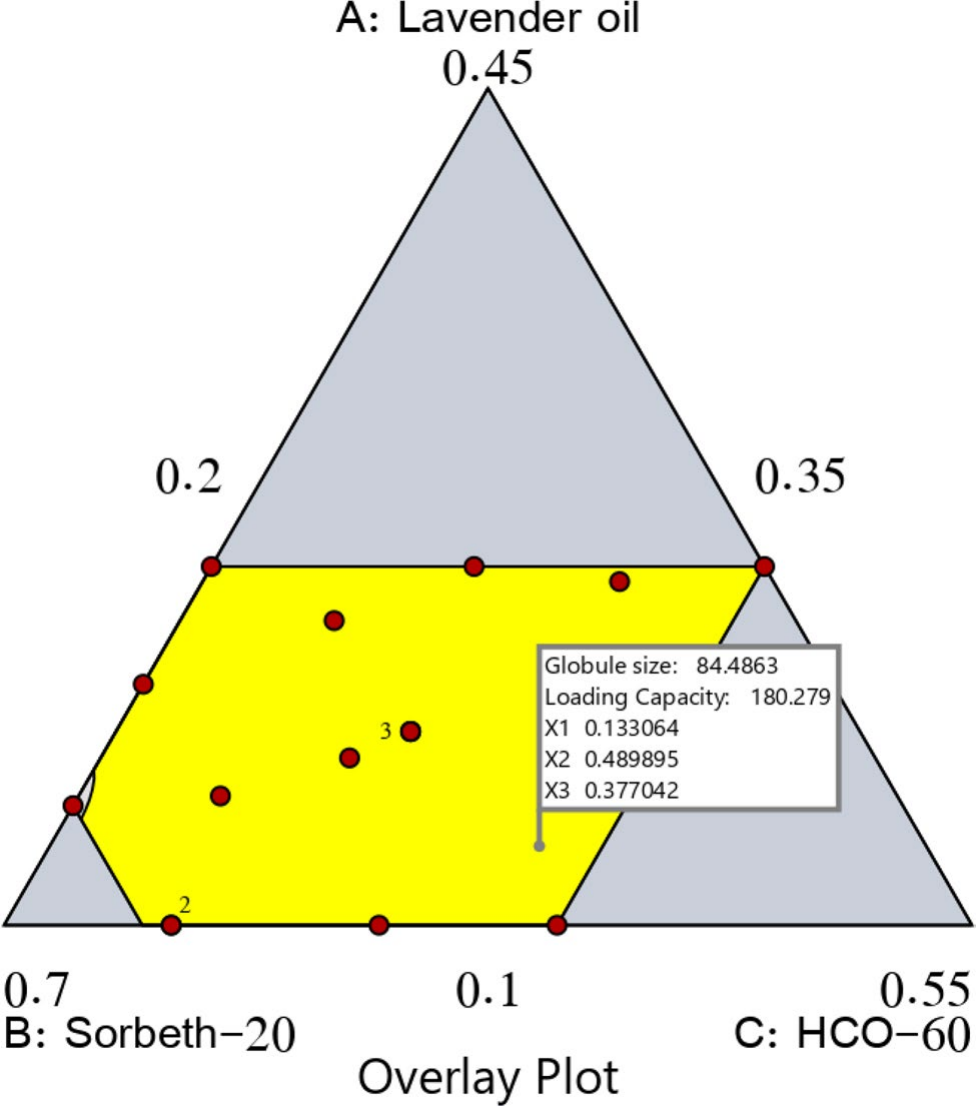
Overlay plot for the optimal ZP-LV-SNEDDS region.

As previously stated, the predicted and adjusted R^2^ values of the dependent variables matched well, and this indicated the ability of the design to predict the capacity. More importantly, the actual and expected ratios recorded for the experimental and predicted responses had a low percentage of error and good residuals; this indicated the lack of curvature in the responses and the validity of the model, as shown in Table 5. Figure 6 presents the overlay plot for the optimal region.

**Table 5.** Composition and actual and predicted responses of the optimal NE formulation.

Factor	Optimal value	Response variable	Actual value	Predicted value	% Prediction error^a^
A: LV oil %	13.3	Droplet size (nm)	87	84.48	0.028
B:Sorbeth-20%	49.0	Drug loading (%)	185	180.28	0.005
C:HCO-60%	37.7				

a Calculated as (Actual – Predicted/Actual) * 100.

### 3.8. *In-vitro* characterization of ZP-LV-SNELTs

The ZP-LV-SNELTs were analyzed to determine different parameters. The weight uniformity of the SNELTs met the pharmacopeia requirement, and the results were 150 ± 2.01 mg. The thickness of the SNELTs was 4.982 ± 0.14. The actual drug content was 9.6 ± 0.24 mg, which is equivalent to 96% ± 2 of ZP content when compared with the theoretical drug content (i.e., 10 mg). These findings complied with the pharmacopeia limits, which state that the drug content of developed tablets should range from 90.0% to 110.0%. The friability of the tablets was less than 1%, which agreed with the pharmacopeia specifications and indicated good mechanical strength. Regarding the disintegration time, the optimized ZP-LV-SNELTs formulation disintegrated within 30 sec, and this indicated the porous nature of the formulation, which enhanced its dissolution. Comparable results were found in the literature (Hosny et al., 2020).

**Figure 7.**
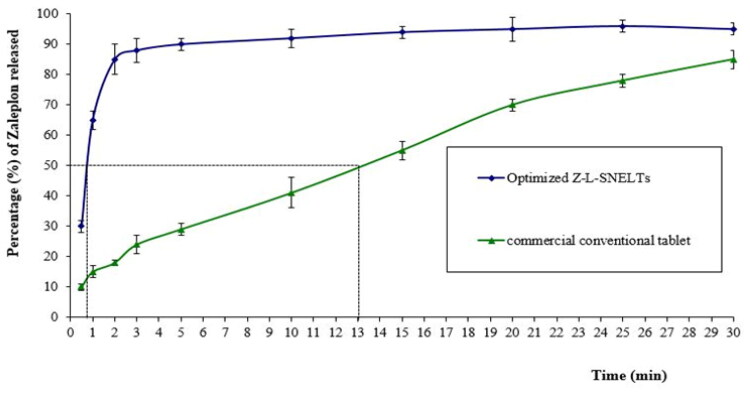
*In-vitro* drug release profiles of ZP from optimal ZP-VL-SNELTs formulation and commercial ZP tablet.

The *in-vitro* dissolution results indicated that 50% of the ZP dose loaded in the optimized ZP-LV-SNELTs formulation was released in simulated saliva fluid with a pH of 6.8 within 45 sec compared with the commercial conventional tablet formulation, which released the same drug amount (50%) after 13 min. The enhancement in ZP release from the developed optimal formulations signified the improvement of ZP dissolution in the nanosized drug delivery system, which offered a large surface area for drug dissolution and release (Rizg et al., 2021). Figure 7 shows the in-vitro drug release profiles of the optimized ZP-LV-SNELTs versus the commercial formulation. The difference in the release time of 50% of loaded ZP dose between the prepared tablets and commercial ones was found to be highly significant (p-value < .0001).

### 3.9. *In-vivo* bioavailability and pharmacokinetic study

**Figure 8.**
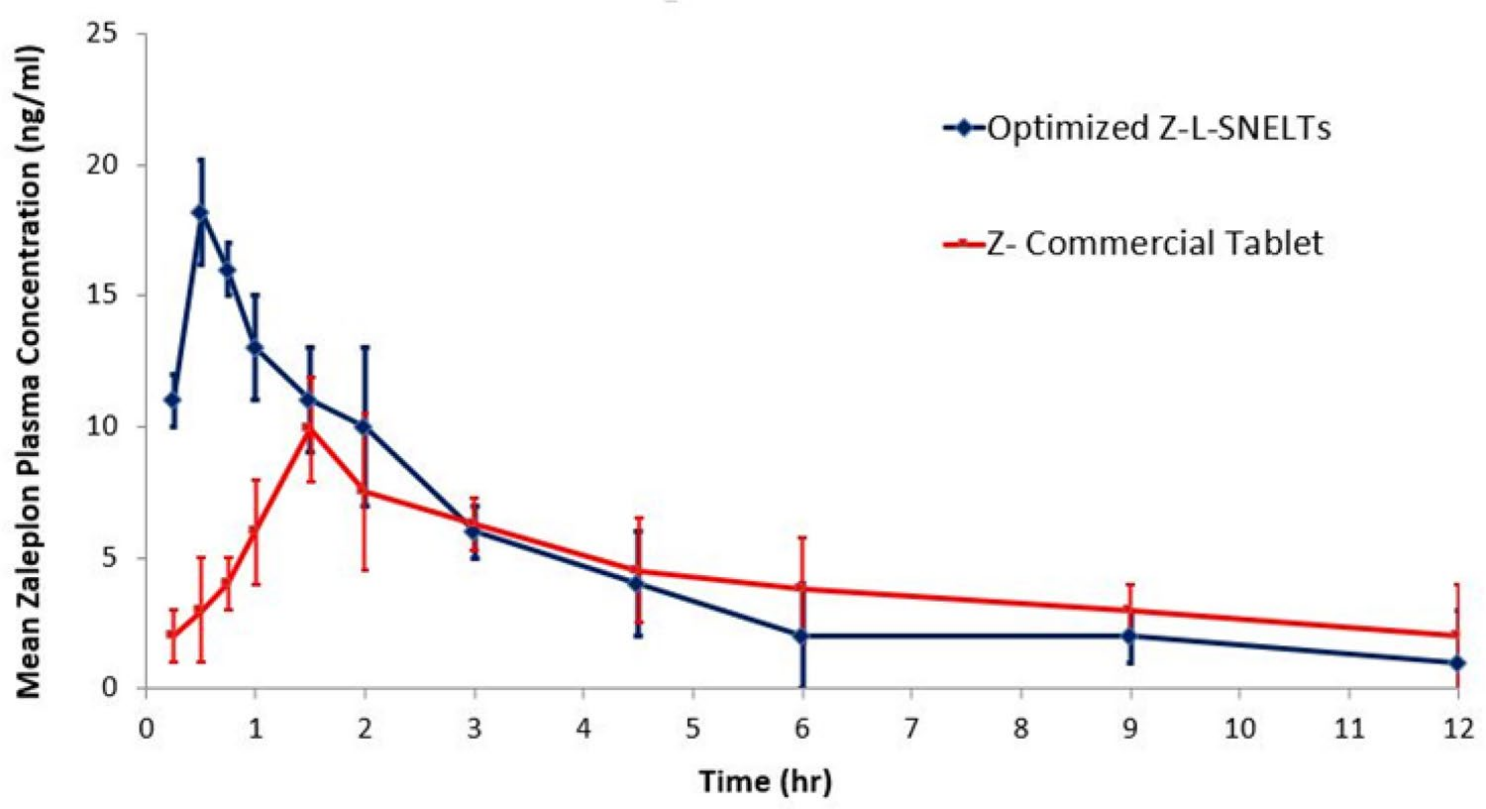
Plasma-concentration time curve of optimized ZP-LV-SNELTs and marketed ZP tablet.

The maximum plasma concentration of ZP (C_max_) and the time required to reach it (T_max_) were obtained directly from the plasma concentration-time curve (Figure 8). The area under the curve (AUC_0→t_) was estimated by the trapezoidal rule. It was found that the C_max_ obtained following ZP-LV-SNELTs administration (18.22 ± 2.35) was remarkably higher than that of the commercial ZP tablet (9.98 ± 1.22) at a *p*-value of less than .05. Additionally, the T_max_ obtained following the optimal ZP-VL-SNELTs administration (0.5 ± 0.25) was considerably lower than that obtained following commercial product administration (1.5 ± 0.25) at a *p*-value of less than .0002. The t_1/2_ for both formulations varied nonsignificantly (1.54 for the test formulation and 1.60 for the standard formulation). The area under the concentration-time curve value, which clarifies the extent of drug absorption, was 835.6 ± 41.33 ng h/mL following administration of the optimal formulation. This was significantly greater than that of the commercial tablets (511.7 ± 32.11 ng h/mL) at a *p*-value of less than .001). Moreover, there was a nonsignificant difference between the MRTs of both the test and standard formulations.

The greater C_max_ and AUC and lower T_max_ obtained after optimal ZP-LV-SNELTs administration could be ascribed to the fast dissolution of that formulation and the greater absorption of drug from it. This may be due to the applied nanosized drug delivery system, which offers a large area for release and better absorption. Similar results were reported in the literature (Khalifa et al., 2019). Such results may be beneficial commercially as it saves a great deal of drug by reducing the required dose per tablet due to the great increase in bioavailability. The prepared formulation is recommended to be clinically investigated in a future study to inspect its efficiency and its drawbacks in humans. Table 6 summarizes the pharmacokinetic parameters of the tested formulations.

**Table 6.** Pharmacokinetic parameters of optimized ZP-LV-SNELTs and marketed ZP tablet.

	Tmax (h)	Cmax (ng/mL)	AUC_0–t_ (ng.h/mL)	K (h-1)	MRT (h)	AUC_0-∞_ (ng.h/mL)
Optimized ZP-LV-SNELTs	0.5 ± 0.25	18.22 ± 2.35	835.6 ± 41.33	0.646 ± 0.13	3.8 ± 0.30	1056 ± 42.1
Marketed zaleplon tablet	1.5 ± 0.25	9.98 ± 1.22	511.7 ± 32.11	0.622 ± 0.10	4.1 ± 0.33	674 ± 36.5

## 4. Conclusion

The developed NE had a high capacity for enhancing the solubility and dissolution of ZP. A pseudoternary phase diagram was constructed to obtain the best levels of LV, Sorbeth-20, and HCO-60 to allocate the most satisfactory NE region for the formulations. The optimal NE had a droplet size of 87 nm with reasonable homogeneity and a drug-loading capacity of 185 mg/mL. The developed ZP-LV-SNELTs had acceptable friability, weight uniformity, and a fast disintegration time, in addition to a 17-fold faster in-vitro release rate, compared with the commercial ZP tablets. Further, the optimized ZP-LV-SNELTs had an increase of approximately 1.6 in drug bioavailability in rabbits compared with the marketed product. In summary, this study highlighted the ability of LV-based self-nanoemulsified tablets to provide better drug solubility and dissolution and, thus, a faster onset of action with better bioavailability. In addition to the high capacity of the oral mucosal delivery to achieve high drug bioavailability thru avoiding first pass metabolism. We recommend studying the prepared formulation clinically in humans as a future prospect of the study.
